# Protocol for the estimation of drinking water quality index (DWQI) in water resources: Artificial neural network (ANFIS) and Arc-Gis

**DOI:** 10.1016/j.mex.2019.04.027

**Published:** 2019-04-29

**Authors:** Majid RadFard, Mozhgan Seif, Amir Hossein Ghazizadeh Hashemi, Ahmad Zarei, Mohammad Hossein Saghi, Naseh Shalyari, Roya Morovati, Zoha Heidarinejad, Mohammad Reza Samaei

**Affiliations:** a Department of Environmental Health Engineering, School of Public Health, Shiraz University of Medical Sciences, Shiraz, Iran; b Department of Epidemiology, School of Health, Shiraz University of Medical Sciences, Shiraz, Iran; c Shahid Beheshti University of Medical Sciences, Tehran, Iran; d Department of Environmental Health Engineering, Faculty of Health, Gonabad University of Medical Sciences, Gonabad, Iran; e Social Determinants of Health Research Center, Department of Health, School of Public Health, Gonabad University of Medical Sciences, Gonabad, Iran; f Department of Environmental Health Engineering, School of Public Health, Sabzevar University of Medical Sciences, Sabzevar, Iran; g Department of Environmental Health Engineering, School of Public Health, Tehran University of Medical Sciences, Tehran, Iran; h Food Health Research Center, Hormozgan University of Medical Sciences, Bandar Abbas, Iran

**Keywords:** Estimation a water quality index in Bardaskan city, Drinking water, WQI, Bardaskan villages, Iran

## Abstract

Drinking water sources may be polluted by various pollutants depending on geological conditions and agricultural, industrial, and other human activities. Ensuring the safety of drinking water is, therefore, of a great importance. The purpose of this study was to assess the quality of drinking groundwater in Bardaskan villages and to determine the water quality index.

Water samples were taken from 30 villages and eighteen parameters including calcium hardness (CaH), total hardness (TH), turbidity, pH, temperature, total dissolved solids (TDS), electrical conductivity (EC), alkalinity (ALK), magnesium (Mg^2+^), calcium (Ca^2+^), potassium (K^+^), sodium (Na^+^), sulphate (SO_4_^2−^), bicarbonate (HCO_3_^−^), fluoride (F^−^), nitrate (NO_3_^−^), nitrite (NO_2_^−^) and chloride (Cl^−^) were analyzed for the purpose for this study. The water quality index of groundwater has been estimated by using the ANFIS. The spatial locations are shown using GPS. The results of this study showed that water hardness, electrical conductivity, sodium and sulfate in 66, 13, 45 and 12.5% of the studied villages were higher than the Iranian drinking water standards, respectively. Based on the Drinking Water Quality Index (DWQI), water quality in 3.3, 60, 23.3 and 13.3% of villages was excellent, good, poor and very poor, respectively.

• Groundwater is one of the sources of drinking water in arid and semi-arid regions such as Bardaskan villages, which monitor the quality of these resources in planning for improving the quality of water resources.
• The DWQI can clearly provide information associated with the status of water quality resources in Bardaskan villages.
• The results of this study clearly indicated that with appropriate selection of input variables, ANFIS as a soft computing approach can estimate water quality indices properly and reliably.
• Some parameters were in the undesirable level is some villages. Therefore, the government should try to improve the chemical and physical quality of drinking water in these areas with the necessary strategies.

## Graphical abstract

**Figure:**
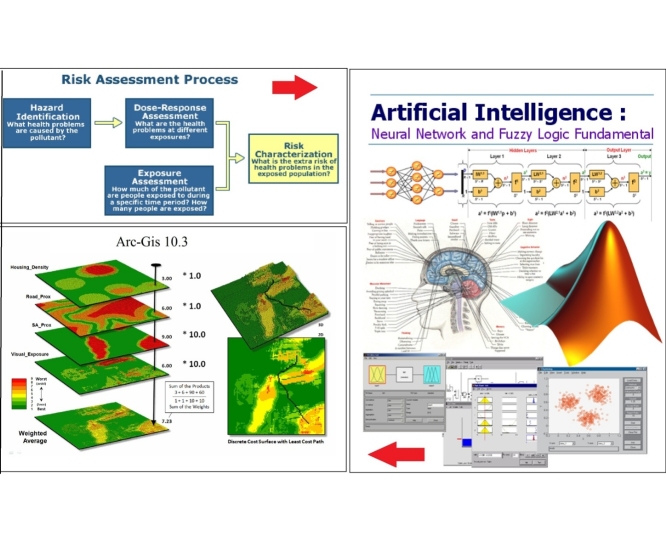

**Specifications Table**

**Table:** 

Subject area:	Environmental Sciences
More specific subject area:	Drinking Water Quality Index (DWQI)
Protocol name:	Estimation a water quality index in Bardaskan city
Reagents/tools:	pH meter (model wtw), turbidity meter (model Hach 50161/co 150 model P2100Hach, USA), spectrophotometer (model DR 5000). Arc-GIS and MATLAB
Experimental design:	The mentioned parameters above, were analyzed according to Standard Methods for the Examination of Water and Wastewater.
Trial registration:	MATLAB:271828 and GIS: 10.4.1
Ethics:	No applicable

## Description of protocol

Clean water is necessary for human communities and generally it is a necessary input to human production and an important tool of economic development [1]. It has a considerable role in social prosperity and the health of human [2,3]. Water quality is dependent on water composition and can be affected by natural process and human activities [4]. Aquifers are important freshwater sources that provide human with water for many purposes such as drinking, agricultural, industrial and recreation [5]. Water resources in many Iranian urban and rural areas face serious threats deriving from groundwater pollution, increasing industrial and agricultural activities coupled with environmental pollution and improper management of all types of wastes [[6], [7], [8], [9]]. After contamination, the restoration of its quality groundwater quality is difficult it usually takes a long time to regain its natural state [10,11]. Consistent and regular monitoring of groundwater quality in a region identifies areas with potential environmental health problems. Recently, water quality indices have been considerably used by many researchers in many nations [[12], [13], [14], [15], [16], [17], [18]]. Drinking Water Quality Index (DWQI) gives a numerical value that shows overall quality of water, by considering the different physico-chemical parameters of water at a certain location and time [[19], [20], [21]]. The distribution map of DWQI in the studied villages are shown using GIS software.

## Materials and methods

### Study area description

**Fig. 1:**
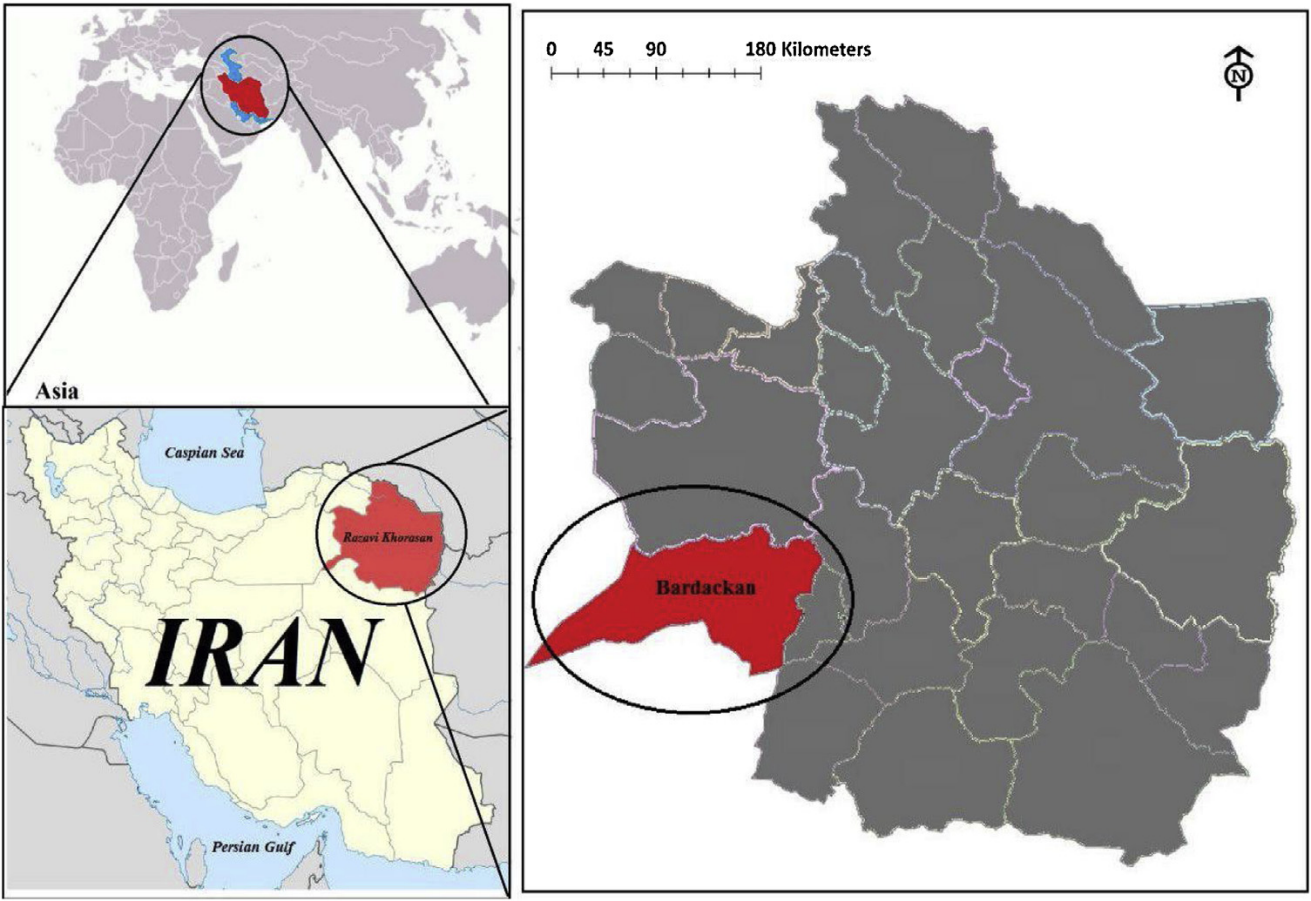
Location of the study area in Bardaskan city, Khorasan Razavi, Iran.

The city of Bardaskan is located in Razavi Khorasan Province, in eastern Iran. The city covers an area of 7664 km^2^, located between 35° 15′N and 57° 58′E. Neighboring cities of the Bardaskan are Sabzevar city (in the North), Khalilabad (in the east), Tabas (in the south) and Semnan (in the west). Bardaskan’s temperature in the hottest summer day is nearly 45 °C and in the coldest winter night is −5 °C and the average annual precipitation is 150 mm. Location of the study area in Bardaskan city in Khorasan Razavi and in Iran is shown in Fig. 1.

### Sample collection and analysis

**Fig. 2:**
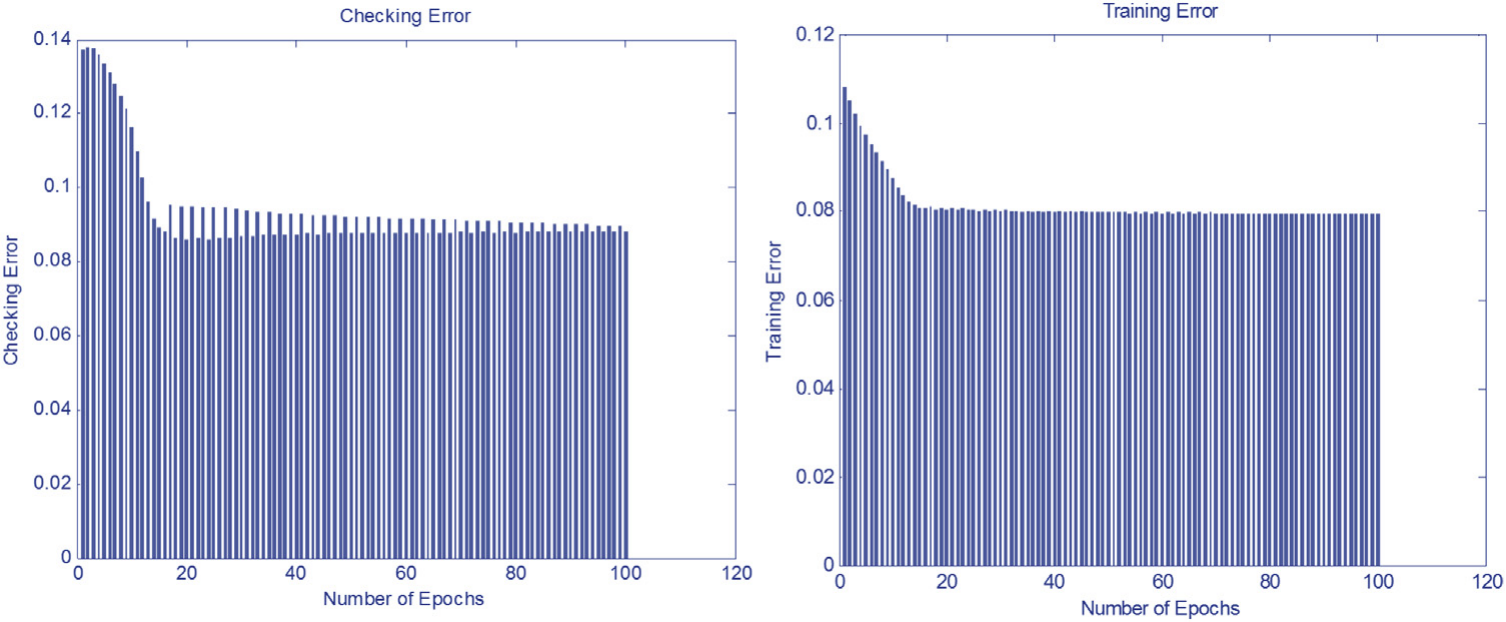
Checking and training errors DWQI for optimization of epochs.

All the chemicals used in this study were of analytical grade and were purchased from the Merck. A total of thirty (30) water samples were taken for main drinking water resources of 30 villages of Bardaskan during 2016–2017. Villages were coded as 1–30. All samples were collected in polyethylene bottles and then transferred to water and wastewater laboratories at temperatures below 4 °C. Eighteen (18) parameters including calcium hardness (CaH), total hardness (TH), turbidity, pH, temperature, total dissolved solids (TDS), electrical conductivity (EC), alkalinity (ALK), magnesium (Mg^2+^), calcium (Ca^2+^), potassium (K^+^), sodium (Na^+^), sulphate (SO_4_^2−^), bicarbonate (HCO_3_^−^), fluoride (F^−^), nitrate (NO_3_^−^), nitrite (NO_2_^−^) and chloride (Cl^−^) were analyzed for the purpose for this study. All water samples were analyzed using standard method for the examination of water and wastewater. Titrimetric method was used for hardness, magnesium, calcium and chloride determination [[22], [23], [24], [25]]. pH was analyzed with pH meter (model wtw, Esimetrwb), EC was determined with Esimetrwb device, turbidity with turbidity meter (model Hach 50161/co 150 model P2100Hach, USA). Fluoride, nitrate and sulfate were also determined by the Hach DR5000 spectrophotometer in the Bardaskan Rural Water and Wastewater Laboratory [25,26]. Finally, the results of water quality in Bardaskan villages were compared with Iran's drinking water standard 1053 [27,28]. Then, in order to determine the water quality in Bardaskan villages, the DWQI was determined according to the following equations (Fig. 2). Firstly, the following equation was used to compute the relative weight [21]:

$$\text{W}\text{i}=\sum \frac{\text{w}\text{i}}{\sum_{\text{i}=1}^{\text{n}}\text{w}\text{i}}$$

Which is in this equation, wi is the relative weight, Wi is the weight of each parameter and n is the number of parameters. Secondly, the quality rating scale for each parameter is calculated by dividing its concentration in each water sample by its respective standards World Health Organization and multiplied the results by 100.

$$\text{q}\text{i}=\left(\frac{\text{C}\text{i}}{\text{S}\text{i}}\right)\times 100$$

Where, qi is the quality rating, Ci is the concentration of each chemical parameter in each sample in mg/L and Si is the World Health Organization (WHO) guideline for each parameter in mg/L according to the WHO, For computing the final stage of DWQI, the SI is first determined for each parameter. The sum of SI values gives the water quality index for each sample.

Si=Wi×qi

DWQI=∑SIi

SIi is the sub-index of ith parameter, and qi is the rating based on concentration of ith parameter and n is the number of parameters [20].

### Modeling by neural-fuzzy systems

Adaptive network-based fuzzy inference (ANFIS), based on the ﬁrst-order Sugeno fuzzy model, was used in this study [29]. This method combines multilayer feed forward back-propagation network and fuzzy inference system and takes the advantages of artificial neural networks and fuzzy logic [30,31]. Over the recent years environmental researchers have utilized this method for several tasks such as prediction, modeling, system control and decision making [32,33]. And for the final analysis of the ANFIS, MATLAB V.20178b software was used. ANFIS as a soft computing approach can estimate water quality indices properly and reliably [34,35].

## Results

**Fig. 3:**
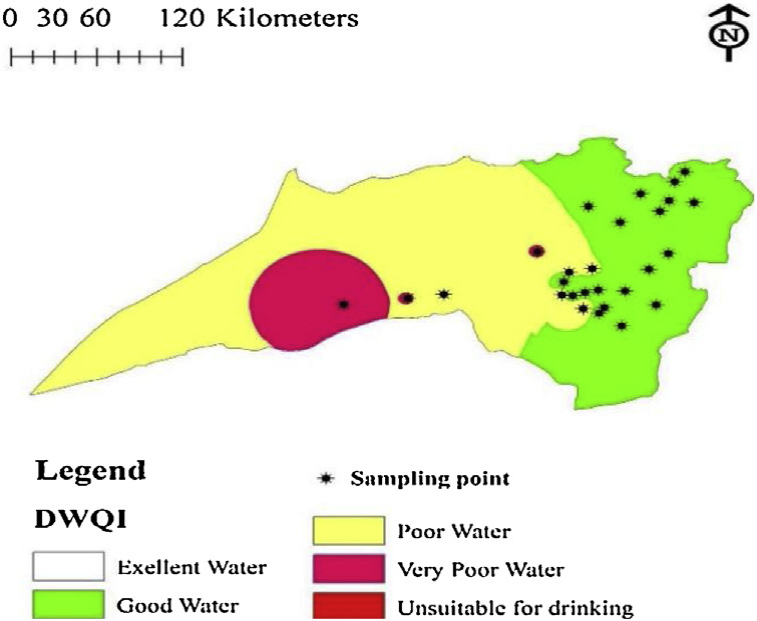
Spatial Distribution Map of Drinking Water Quality Index.

Results of studied parameters including hardness, pH, turbidity, temperature, total dissolved solids and electrical conductivity in water samples of Bardaskan villages are shown in Table 1. Cations and anions measured in these areas are also shown in Table 2. The comparison of quality of water resources in Bardaskan villages with Iran's drinking water standard 1053 are listed in Table 3. The water quality index was used to compare the quality of drinking water resources in Bardaskan villages (Table 5). The classification of water quality is given in Table 4. Also, the results of drinking water quality in Bardaskan villages based on the water quality index are shown in Table 6. Table 7 show predicting performance in different steps of ANFIS. Spatial Distribution Map of Drinking Water Quality Index is shown in Fig. 3.

**Table 1:** Physico-chemical parameters of water resources of villages of Bardaskan city during.2016–2017.

Village code	CaH (mg/L as CaCO_3_)	TH (mg/L as CaCO_3_)	Turbidity (NTU)	pH	T (°C)	TDS (mg/L)	EC (μmhos/cm)	ALK (mg/L as CaCO_3_)
1	28	68	3	8.38	22.3	698	1125	222
2	32	80	1.09	8.27	21.2	556	897	180
3	36	92	0.21	8.24	22.6	642	1036	169
4	36	80	0.58	8.23	22.7	575	928	169
5	40	84	4.58	8.32	22.5	613	989	147
6	28	48	0.63	8.39	22.4	478	771	160
7	42	92	0.33	8.29	20.8	815	1314	188
8	148	440	0.23	7.96	20.2	811	1308	357
9	48	92	0.26	8.33	20.8	843	1359	192
10	170	620	6.3	8.02	20	1414	2280	211
11	110	232	0.42	8.13	20.2	2864	4620	162
12	64	148	0.34	8.26	20.9	1063	1714	102
13	32	64	0.24	8.33	21.4	753	1214	214
14	64	104	0.28	8.13	21.3	1045	1686	274
15	88	116	0.23	8.04	26.2	307	495	160
16	184	300	0.53	7.65	25.9	725	1170	293
17	152	280	0.28	7.8	25.8	586	945	278
18	124	156	0.3	7.89	25.8	358	577	196
19	136	200	0.22	7.93	25.8	455	734	218
20	120	204	0.26	8.03	25.8	650	1049	271
21	170	270	0.46	7.88	25.5	678	1094	432
22	112	176	0.25	8.15	25.6	487	785	229
23	260	440	0.56	7.81	22.7	1662	2680	188
24	60	112	0.47	8.2	23.1	1037	1672	331
25	36	84	0.48	8.31	23.1	596	962	142
26	40	96	0.47	8.32	23	627	1012	124
27	32	56	0.33	8.14	22.7	443	715	139
28	24	96	0.6	8.28	22.6	520	839	192
29	32	124	0.18	8.47	21.5	963	1554	237
30	264	444	0.27	7.82	12.8	1810	2920	177
Mean	90.40	179.93	0.81	8.13	22.57	835.80	1348.13	211.80
Max	264.00	620.00	6.30	8.47	26.20	2864.00	4620.00	432.00
Min	24.00	48.00	0.18	7.65	12.80	307.00	495.00	102.00
SD	69.14	142.45	1.38	0.21	2.71	521.40	840.99	72.69

**Table 2:** Cations and anions measured in water resources of villages of Bardaskan city during the years 2016–2017.

Village code	Mg^2+^ (mg/L)	Ca^2+^ (mg/L)	K^+^ (mg/L)	Na^+^ (mg/L)	SO_4_^2−^ (mg/L)	HCO_3_^−^ (mg/L)	F^−^ (mg/L)	NO_3_^−^ (mg/L)	NO_2_^−^ (mg/L)	Cl^−^ (mg/L)
1	**96**	11.2	1	228	173	271	0.8	11.59	0.014	106
2	11.52	12.8	1.3	172	127	220	0.71	0	0	77
3	13.44	14.4	1.6	195	157	206	0.58	6.72	0.02	115
4	10.56	14.4	1.5	180	147	206	0.54	5.89	0.006	86
5	10.56	16	1.4	187	185	179	0.55	6.07	0.003	93
6	4.8	11.2	1.1	150	113	168	0.67	5.52	0	60
7	12	16.8	1	250	211	229	0.67	9	0.048	148
8	70.08	59.2	4.5	120	189	436	0.29	6.72	0.003	95.06
9	10.56	19.2	1	260	174	234	0.57	9.02	0.008	179
10	108	68	3	250	296	257	0.33	22.45	0.006	451
11	29.28	44	5	950	704	198	0.88	11.04	0.004	926
12	20.16	25.6	1	308	428	124	0.64	0.92	0.003	175
13	7.68	12.8	1	246	169	261	0.86	14.35	0.006	121
14	9.6	25.6	1	356	206	334	1.03	18.58	0.003	221
15	6.72	35.2	0.4	62	43.58	195	0.31	18.95	0.003	22.54
16	27.84	73.6	2	139	124	357	0.49	17.2	0.007	128
17	30.72	60.8	1.1	96	145	339	0.41	12.05	0.01	46.06
18	7.68	49.6	0.5	67	48.3	239	0.2	15.92	0.003	27.44
19	15.36	54.4	0.4	81.5	65.1	266	0.39	46.55	0.006	44.1
20	20.16	48	1.5	148	152	331	0.5	22.36	0.004	75.46
21	24	68	1.2	138	75.6	527	0.49	13.43	0.001	51.94
22	15.36	44.8	1	102	96	279	0.41	26.13	0	46.06
23	43.2	104	2	450	567	229	0.68	68.24	0.002	416
24	12.48	24	1	355	263	404	0.59	24.38	0.006	171
25	11.52	14.4	1.4	181	209	173	0.46	5.89	0	88.2
26	13.44	16	1.6	1881	256	151	0.49	8.98	0	92.12
27	5.76	12.8	1.3	144	135	170	0.63	8.19	0	62.72
28	17.28	9.6	1.8	156	147	234	0.61	9.57	0.004	64.68
29	220.8	12.8	2	314	207	233	0.57	24.25	0.017	198
30	43.2	105.6	4	480	586	216	0.73	77.37	0.008	437
Mean	30.992	36.16	1.62	288.217	213.286	255.533	0.56933	17.569	0.0065	160.813
Max	220.8	105.6	5	1881	704	527	1.03	77.37	0.048	926
Min	43.8528	27.657	1.1158	346.28	158.807	89.4523	0.18515	17.7	0.00926	183.947
SD	43.8528	27.657	1.1158	346.28	158.807	89.4523	0.18515	17.7	0.00926	183.947

**Table 3:** Comparison of physicochemical quality of water resources of villages in Bardaskan city with the standard of drinking water of Iran during the years 2016–2017 [3,4,8,20].

Parameter	1053IR Standard		Percentage of villages		
	Desirable	Limit	Desirable	Limit	More than standard
pH	6.5–8.5	6.5–9	100	–	–
TDS (mg/L)	500	1500	70	20	10
CL^−^ (mg/L)	250	400	–	88	12
SO_4_^2−^ (mg/L)	250	400	10	77.5	12.5
NO_3_^−^ (mg/L)	–	50	–	94	6
NO_2_^−^ (mg/L)	–	3	100	–	–
Ca^2+^ (mg/L)	300	400	100	–	–
Mg^2+^ (mg/L)	30	150	16.5	83.5	–
Na^+^ (mg/L)	200	200	–	55	45
F^−^ (mg/L)	0.5	1.5	65	35	–
TH (mg/L as CaCO_3_)	200	500	30.5	35	66
Turbidity (NTU)	<1	5	96.6	–	3.4
EC (μmhos/cm)	1500	2000	13	74	13

**Table 4:** Water quality classification ranges and types of water based on DWQI values [17].

DWQI value	Class	Explanation
<50	Excellent	Good for human health
50–100	Good	Fit for human consumption
100–200	Poor	Water not in good condition
200–300	Very poor	Need attention before use
>300	Inappropriate	Need too much attention

**Table 5:** Relative weight of chemical of physico-chemical parameters [1,9,17,21].

Number	Factor	Factor Weight	WHO Standard
1	K^+^	2	12
2	Na^+^	3	200
3	Mg^2+^	2	50
4	Ca^2+^	3	75
5	HCO_3_^−^	2	500
6	NO_3_^−^	5	45
7	NO_2_^−^	5	3
8	SO_4_^2−^	4	250
9	CL^−^	3	250
10	F^−^	4	1.5
11	TH	3	100
12	EC	3	1500
13	TDS	5	500
14	pH	3	6.5–8.5

**Table 6:** Results of Drinking Water Quality Index (DWQI) of Bardaskan villages during 2016–2017.

Village number	DWQI	Water quality rating	Village number	DWQI	Water quality rating
1	87.10	Good	16	104.39	Poor
2	63.93	Good	17	90.08	Good
3	73.12	Good	18	57.13	Good
4	66.29	Good	19	69.44	Good
5	70.45	Good	20	86.18	Good
6	53.82	Good	21	93.30	Good
7	86.09	Good	22	69.83	Good
8	128.21	Poor	23	206.96	Very poor
9	87.99	Good	24	106.67	Poor
10	203.38	Very poor	25	70.08	Good
11	278.04	Very poor	26	162.14	Poor
12	115.51	Poor	27	54.13	Good
13	76.76	Good	28	64.32	Good
14	105.88	Poor	29	132.80	Poor
15	48.31	Excellent	30	217.18	Very poor

**Table 7:** Predicting performance in different steps of ANFIS.

Index	RMSE^a^	R^2^	MAE^b^	MSE^c^
DWQI				
Train	2.34	0.0875	1.23	4.59
Check	2.33	0.1164	1.24	4.81

DWQI-Cold				
Train	2.87	0.1839	1.22	3.61
Check	2.89	0.2808	0.923	1.09

DWQI-warm				
Train	3.69	0.1159	1.14	1.09
Check	3.71	0.2028	1.09	4.03

a Root mean squares error.

b Mean absolute error.

c Mean squared error.

## Conclusions

It is important to have exact information about main drinking water parameters in order to find the source of pollution. DWQI is a good platform for proper assessment, management and protection of water resources in an area. The results showed that the values of SO_4_^2−^, NO_3_^−^, TH, and Na^+^ were above the WHO and local standards in the study areas. Based on the Drinking Water Quality Index (DWQI), water quality in 3.3, 60, 23.3 and 13.3% of villages was excellent, good, poor and very poor. Therefore, regular monitoring is essential in order to ensure safe drinking water to consumers in the studied areas at the optimum level according to the WHO and national limits, especially in villages with poor and very poor water quality status. As groundwater is the main source of water by local people in Bardaskan villages, applying more audits by governmental offices on water withdrawal and its quality issues is suggested.

## Conflict of interest

The authors declare have no any conflict of interests.
